# Clinical Importance of Wnt5a in the Pathogenesis of Colorectal Cancer

**DOI:** 10.1155/2021/3136508

**Published:** 2021-09-23

**Authors:** Mehran Pashirzad, Thozhukat Sathyapalan, Amirhossein Sahebkar

**Affiliations:** ^1^Department of Medical Biochemistry, School of Medicine, Mashhad University of Medical Sciences, Mashhad, Iran; ^2^Department of Academic Diabetes, Endocrinology and Metabolism, Hull York Medical School, University of Hull, Hull HU3 2JZ, UK; ^3^Biotechnology Research Center, Pharmaceutical Technology Institute, Mashhad University of Medical Sciences, Mashhad, Iran; ^4^Applied Biomedical Research Center, Mashhad University of Medical Sciences, Mashhad, Iran; ^5^School of Pharmacy, Mashhad University of Medical Sciences, Mashhad, Iran

## Abstract

Wnt5a is one of the potent signaling molecules that initiates responses involved in cancer through activation of both canonical and noncanonical signaling cascades. Wnt5a both directly and indirectly triggers cancer-associated signaling pathways based on the cancer type. In colorectal cancer (CRC), altering Wnt5a expression can influence several cellular processes of tumor cells, including proliferation, differentiation, migration, invasion, and metastasis. This review summarizes the molecular mechanisms and clinical importance of Wnt5a in the pathogenesis of CRC for better understanding the pathogenesis and its potential role as a prognostic marker and as an appropriate therapeutic target in the treatment of this disease in the future.

## 1. Introduction

Wnt ligands are a large family of glycoproteins that initiate their signaling functions through binding to a member transmembrane of G-protein-coupled frizzled (Fzd) receptor and eventually activation of scaffolding protein disheveled (Dvl) [1, 2]. Wnt proteins are generally functionally divided into canonical and noncanonical Wnt ligands, regulating several intracellular processes, including proliferation, migration, differentiation, and polarity, regulating both canonical and noncanonical Wnt *β*-catenin signaling cascades [3, 4].

## 2. Canonical and Noncanonical Wnt Signaling

In canonical Wnt *β*-catenin signaling, the expression of target genes is regulated by the presence and absence of Wnt ligands [5, 6]. In the absence of a Wnt protein, *β*-catenin is degraded and ubiquitinated by key components of the destruction complex, including glycogen synthase kinase 3*β* (GSK3*β*), adenomatous polyposis coil (APC), and Axin [7, 8]. Following binding Wnt ligands to the Fzd receptor, the destructive complex is inactivated, leading to translocation of *β*-catenin into the nucleus where it tightly binds to specific transcription factors such as T cell factor/lymphoid enhancer-binding factor (TCF/LEF) to stimulate expression of target genes involved in cell proliferation and differentiation [9, 10].

In contrast to the canonical Wnt *β*-catenin signaling, noncanonical Wnt signaling, independent of *β*-catenin stabilization, regulates expression of Wnt noncanonical ligand and several cellular processes, including through activation of both Wnt/Ca2^+^ and planar cell polarity (PCP) [11, 12]. Following the binding of Wnt noncanonical proteins to the Fzd receptors and activation of Dvl, the PCP signaling pathway is triggered [13]. Regarding the activated Dvl in stimulated cells, other intracellular mediators of the PCP pathway, including Rho, Rac, Rho-associated kinase (ROCK), and c-Jun N-terminal kinase (JNK), are also activated, respectively [14, 15]. Following activation of the PCP pathway, this complicated signaling plays a key role in regulating actin polymerization and myosin activation [16, 17]. In contrast to the PCP pathway, the Wnt/Ca2^+^ signaling is also triggered by the interaction between noncanonical Wnt ligands such as Wnt5a and the Ror2 receptor, which leads to the activation of phospholipase C- (PLC-) induced inositol 1,4,5 triphosphate (IP_3_), and 1,2 diacylglycerols (DAG) production forms membrane-bound phosphatidyl 4,5-bisphosphate [18]. Then, activated IP3 releases Ca2+ from the endoplasmic reticulum (ER), which eventually can activate transcription factors including calcium/calmodulin-dependent protein kinase ǀǀ- (CaMKǀǀ-) induced nuclear factor-*κ*B (NF-*κ*B) and protein phosphatase calcineurin- (Cn-) induced nuclear factor associated with T cells (NFAT) [19, 20]. Eventually, this complex pathway plays an important function in regulating cell adhesion, cytoskeleton renewal, migration, and tissue separation by overactivating NFAT and NF-*κ*B [21].

According to the reciprocal relationship between noncanonical signaling and expression of noncanonical Wnt proteins, Wnt5a also activates noncanonical signaling cascades via binding to several receptors, including receptor tyrosine kinase-like orphan receptor 2 (Ror2) and Fzd receptors such as Fzd-4 [22]. Like noncanonical Wnt signaling, the canonical Wnt *β*-catenin is also activated by Wnt5a [23]. In addition, Wnt5a activates the canonical Wnt signaling via binding to Fzd-4 [24, 25]. In line with this, double-labelling immunofluorescence staining showed that Wnt5a could also activate the Wnt *β*-catenin signaling through translocation of *β*-catenin into the nucleus overexpression Fzd-4 and low-expression of Ror-2 in orthodontic tooth movement (OTM) [26]. In further support of the activator effect of Wnt5a on the canonical Wnt *β*-catenin signaling, it has been shown that Wnt5a promotes cyclin D1 through transactivation of ErbB1 and provoking the *β*-catenin/TCF pathway in Wnt-expressing HC11 mammary cells [27]. In contrast to the activator effect of Wnt5a on Wnt *β*-catenin signaling, Wnt5a is also able to inhibit canonical Wnt signaling via binding to the specific receptors, *β*-catenin degradation, and loss of Wnt *β*-catenin target genes expression [28, 29]. In support of the inhibitory function of Wnt5a, it has been reported that Wnt5a, independent of phosphorylation of GSK3*β*, can also inhibit canonical signaling pathways by *β*-catenin degradation and low-expression of Wnt3a in HEK 293 cell lines [30]. In a study, the inhibitory effect of Wnt5a on Wnt *β*-catenin signaling through binding to the Ror2 receptor, downregulation of *β*-catenin, and reducing Wnt3a-mediated SuperTOPFlash (STF) luciferase activation has been proved in a dose-dependent manner of Wnt3a in 293 cells [24].

Consistent with these results, there are generally several factors involved in dysregulation of cellular processes-induced initiation to cancer progression, including genetic and epigenetic alters, which led to activating canonical and noncanonical Wnt signaling aberrantly [31]. In line with this, it has been determined that a wide range of genetic changes of components of the Wnt signaling pathway, including Wnt ligands, Fzd receptors, Dvl, GSK 3*β*, Axin, CK1*α*, APC, and *β*-catenin, are effectively considered as an oncogene and tumor suppressor which could be an appropriate therapeutic target in cancers [9]. One of the most effective noncanonical Wnt ligands which can significantly antagonize canonical Wnt ligand functions and eventually suppress Wnt *β*-catenin signaling or canonical Wnt signaling is Wnt5a [32]. Recent findings demonstrate that Wnt5a noncanonical ligand, as a tumor suppressor and oncogenic agent, can promote and inhibit tumor processes through canonical and noncanonical Wnt signaling in many common cancers such as colorectal breast, prostate, ovarian, bladder, and lung cancer [33, 34].

## 3. Wnt5a Signaling and Cancer

Tumor suppressor and oncogenic effects of Wnt5a noncanonical ligands have been shown to be mediated by targeting processes related to cellular senescence, cancer stem cells, chemotherapy resistance, tumor microenvironment cells, cancer-associated inflammation, epithelial-mesenchymal transition (EMT), metastasis, cell proliferation, cell invasion, and cell migration in a wide range of various cancer cell lines [34]. Moreover, it has been demonstrated that Wnt5a is an auto and paracrine molecule that stimulates oncogenic and tumor suppressor signaling associated with cancer type. On the other hand, expression levels of Wnt5a determine its function as a tumor suppressor or oncogene in specific cancer types. In line with this, it has been shown that loss of Wnt5a expression, as a tumor suppressor, significantly correlates with stimulation of progression and metastasis of breast cancer cells and reduction of disease-free survival and overall survival [35]. In contrast, increased expression of Wnt5a-induced expression of activated leukocyte cell adhesion (ALCAM) effectively stimulates expression of migratory capacity of estrogen receptor- (ER-) positive breast cancer, which leads to enhance clinicopathologic factors involved in breast cancer, including lymph node metastasis, nuclear grade, and lymphatic invasion [36]. In further support to the tumor suppressor effects and decreased expression of Wnt5a in breast cancer, it has been implicated that loss of Wnt5a expression directly correlates with advancing TNM, decreasing survival and triple-negative status in both sporadic and heredity breast cancer tissues [37]. In contrast with these results, overexpression of Wnt5a in prostate cancer cell lines such as PC3 cells can significantly induce cell apoptosis and reduce cell proliferation and migration as well as modulate local tumor growth and tumor growth in the bone microenvironment, suggesting that Wnt5a can act as a tumor suppressor gene both in vivo and in vitro [38].

The presence of epigenetic changes of Wnt5a, including methylation, in different stages of some cancers such as colorectal cancer (CRC) has shown that aberrant methylation levels of Wnt5a are not significantly correlated with clinicopathological characteristic including maximal tumor size, tumor extent, tumor site, histology, and Dukes stage [39]. In contrast to this result, abnormal levels of Wnt5a methylation-induced loss of Wnt5a expression significantly correlates with the presence of the lymph node and advanced TNM stage, suggesting that Wnt5a acts as a tumor suppressor gene in patients with CRC [40]. In the current review, we summarize molecular mechanisms and clinical importance of Wnt5a in the pathogenesis of CRC to better understand its potential functions both as a prognostic and an appropriate therapeutic target in the treatment of this disease.

## 4. Wnt5a as a Tumor Suppressor and Therapeutic Target in CRC

Based on the findings of recent studies and the available evidence on the suppressive effect of Wnt5a on types of cell lines and tumor tissue of CRC, this protein can be considered a key therapeutic target in inhibition of CRC progression [41]. The epigenetic modification-induced regulation of expression levels of Wnt5a could be a factor to determine the inhibitory effect of Wnt5a in CRC cells [42, 43]. It has been demonstrated that ectopic expression of Wnt5a in methylation-stimulated silencing Wnt5a CRC cells inhibits tumor cell clonogenicity via downregulation of *β*-catenin protein levels and inactivation of Wnt *β*-catenin signaling [44]. To determine the inhibitory effect of Wnt5a on primary colon cancer, the ectopic expression model indicated that overexpression of Wnt5a induced by the ectopic model inhibits cell proliferation and metastasis via suppression of epithelial-mesenchymal transition (EMT), canonical Wnt, and overactivation of Wnt/Ca2+ signaling in HCT116 cell line [45]. Regarding the lower Wnt5a expression induced by methylation in CRC cells, this ligand negatively correlates with chemotherapy drugs such as 5-fluorouracil (5-FU) inhibitory effects. Findings of one study showed that the inhibitory effect of 5-FU on siRNA-induced knockdown Wnt5a expression in unmethylated LoVo and SW480 cells is higher than overexpressed Wnt5a in hypermethylated HCT116 and SW620 cells, suggesting that loss of expression of Wnt5a can have a suppressive effect of 5-FU in CRC cells [46]. The relationship between levels of Wnt5a methylation and intake of vitamin D in patients with CRC decreased levels of Wnt5a methylation induced by vitamin D that inhibit cell proliferation, suggesting that vitamin D intake conversely affect the hypermethylation of Wnt5a [47]. In another study, it has been reported that genistein, a member of the soy isoflavones family, suppresses cell proliferation by decreasing CpG island methylation of Wnt5a and overexpression Wnt5a in the SW1116 cell line [48]. In further support of the loss of Wnt5a expression induced by histone modification of promoter regions in CRC cells, various research findings demonstrated that histone deacetylase (HDAC) inhibitors, including trichostatin A (TSA) and sodium butyrate (NaBt), stimulate mRNA and protein expression of Wnt5a by increasing acetylation levels of histones in its promoter region including H3Ac, H4Ac, and H3K4me2 in highly metastatic human CRC cell line SW620 in comparison with nonmetastatic human CRC cell line SW480 [49]. Since the levels of Wnt5a methylation are usually decreased in the SW480 cell line, the expression levels of Wnt5a are reduced by increasing hypermethylation of H3K27 of Wnt5a promoter induced by activation of enhancer zeste homolog 2 (EZH2) in transforming growth factor *β*- (TGF*β*-) treated SW480 cells [50].

In addition to the loss of Wnt5a expression-determining epigenetic modifications factor, Wnt5a-stimulates overexpression of tumor suppressor genes. The factors involved in the overexpression of Wnt5a also demonstrate the inhibitory function of Wnt5a in CRC cells. In line with this, treatment of CRC cell lines including HT-29 and Caco-2 with recombinant Wnt5a (rWnt5a) or Foxy-5, a Wnt5a mimicking peptide, shows that this recombinant can inhibit cell proliferation and stimulate cell differentiation through overexpression of a tumor suppressor enzyme in prostaglandin E2 catabolism named 15-hydroxyprostaglandin dehydrogenase (15-PGDH) and reduction of Wnt *β*-catenin signaling [51]. To stimulate expression levels of Wnt5a in HT-29 cell line using the colonic extracellular calcium-sensing receptor (CaSR), results of a study demonstrated that overexpression of Wnt5a induced by CaSR suppresses cell proliferation via overexpression of Ror2 and absentia homolog 2 (Siah2) as well as inactivation of Wnt *β*-catenin signaling [52]. Since the CaSR could be modulated by the increased expression of Ror2 and Wnt5a secretion; lipopolysaccharide (LPS), when added to CaSR-HEK cells, RAW264.7 murine macrophage cell lines, or HT-29 cells, stimulates tumor necrosis factor-*α* (TNF-*α*) and CaSR. Following CaSR activation, expression of Wnt5a is significantly increased, which leads to the reduction of TNF receptor 1 (TNFR1) and TNF-*α*-stimulated Wnt3a canonical signaling expression through overexpression of Ror2 in HT-29 CRC cell lines [53]. Regarding the inhibitory role of Wnt5a in suppressing CRC cell proliferation, this ligand is essential for the regeneration of epithelial cells crypts during homeostasis. In line with this, it has been reported that Wnt5a can potentially suppress cell proliferation of injured epithelial cells via activation of TGF-*β* signaling [54]. In further support of the inhibitory role of Wnt5a, it has been indicated that this noncanonical ligand is suppressed by several secretive and regulator mediators such as microRNA 567-5p (miR-567-5p) in CRC, suggesting that these factors determine the function of Wnt5a tumor suppressor. Consistent with these results, the results of RT-qPCR and Western blotting study showed that transfection of miR-567-5p mimic into the CRC cells led to downregulate Wnt5a expression and upregulate levels of *β*-catenin and Wnt *β*-catenin target genes involved in cell proliferation. Conversely, following transfection of pcDNA-Wnt5a and XAV-939 inhibitor into the SW480 cells suppresses cell viability, migration, and invasion of SW480 cells via inhibition of miR-567-5p activity and inactivation of Wnt *β*-catenin signaling, respectively [55]. Like miR-567-5p, miR-26b is an essential regulator during the metastasis process, expressed significantly in CRC cell lines Caco-2 and DLD1. In tumor samples from patients, lymphatic metastasis was determined by real time-polymerase chain reaction (RT-PCR). Since the tumor suppressors such as phosphatase and tensin homolog (PTEN) and Wnt5a have a highly conserved binding site for miR-26b, transfection of miR-26b mimic into the CRC cell lines stimulates metastasis of CRC cells via increasing EMT and downregulation of Wnt5a and PTEN [56].

In several recent studies, the association between the loss of Wnt5a expression and clinicopathological features in the different stages of CRC has been examined. Consistent with the histone modification-induced hypermethylation of Wnt5a, it has been determined that downregulation of Wnt5a has a positive correlation with advanced TNM stages of III and IV as well as lymph node invasion in CRC tissues [40]. On the other hand, to assess the prognostic role of Wnt5a methylation and tumor subtypes, it has been observed that Wnt5a methylation has significantly associated with increasing microsatellite instability (MSI) tumors features, including one mismatch repair protein, MLH1 methylation, proximal location, mucinous histological type, and female gender [57]. Cheng's study showed that low expression of Wnt5a has no association with age, gender, tumor size, histological differentiation, and recurrence. In contrast, it has significantly correlated with poor overall survival in the metastatic group [45]. In a study, it has been reported that hypermethylation of the Wnt5a promoter has been significantly associated with enhancing tumor stage, which is mediated by increasing DNMT1 activity in CRC tissue [58].

## 5. Oncogenic Role and Therapeutic Target of Wnt5a Signaling in CRC

In contrast to the inhibitory effect of Wnt5a-determining factors, including epigenetic modification-induced methylation of Wnt5a promoter, factors-stimulated overexpression of Wnt5a, and Wnt5a-induced overexpression of tumor suppressor genes, the stimulatory effect of Wnt5a on CRC samples has also been examined in several studies. Regarding the oncogenic effect of Wnt5a, this protein can potentially be influenced on CRC cells mediated by specific cells, including tumor-associated macrophages (TAMs). In line with this, findings of a study showed that Wnt5a expressed by TAMs stimulate tumor cell proliferation and migration of CRC cells, including HCT116 and DLD1, and recruited macrophage infiltration through enhancing the CaMKǀǀ-ERK signaling pathway [59]. According to the results of the previous study, the autocrine effect of Wnt5a on TAMs leads to the promotion of CRC tumor growth. Mechanistically, the autocrine activity of Wnt5a-stimulated M2 polarization of TAMs, as a TAM subtype, provokes cell proliferation, migration, and invasion of CRC cells, including HCT116 and DLD1, though increasing secreted interleukin-10 (IL-10) in TAM cells which is mediated by overactivation of CaMKǀǀ-ERK1/2-STAT3 signaling pathways [60]. Since the expression levels of Wnt5a is increased in CRC cells, this ligand can progressively affect the different cellular processes of the tumor cells, including proliferation, differentiation, migration, and invasion. In an inducible Wnt5a transgenic mouse model has been observed that Wnt5a administration stimulates adhesion site and the directional migration and invasion of CRC cells [61].

Consistent with the regulatory and secretory factors in the tumor microenvironment region, a member of the transient receptor potential channel (TrpC) family named TrpC5 forms a receptor to activate the nonselective Ca^2+^ channel. The TrpC5 is highly expressed in CRC cells; this permeable Ca^2+^ channel can play a crucial role in regulating the differentiation of colon tumor cells. It has been demonstrated that TrcP5-expressing poorly differentiated CRC cell lines including SW480, HT-29, SW620, DLD1, HCE8693, and RKO can upregulate Wnt5a and cytoplasmic *β*-catenin expression that finally lead to the decreasing differentiation and enhancing stemness of CRC cells [62]. A regulator factor highly expressed in intestinal epithelial cells (IECs) in tumor microenvironment named calcium/calmodulin-dependent protein kinase ǀǀ gamma (CAMK2*γ*), which is known as a downstream target of Wnt5a noncanonical signaling induced by dextran sodium sulfate- (DSS-) induced colitis-associated cancer (CAC). It has been observed that DSS promotes inflammatory signals and injuring IECs via overexpression of Wnt5a noncanonical ligand and receptors and coreceptors of Wnt5a, including Fzd-2, -5, and Ror2. Following overexpressed Wnt5a and its receptor/coreceptor in IECs, Wnt5a significantly increases the phosphorylation of CAMK2*γ*, which eventually leads to the stimulation of survival and proliferation of colonic epithelial cells through overactivation of epithelial signal transducer and activator of transcription 3 (STAT3) signaling during CAC development [63]. Another regulator factor, as a transcription factor associated with cell proliferation, is named forkhead box transcription factor M1 (FOXM1) which play a crucial role in tumorigenesis via regulating expressed target genes in dendritic cells (DCs) of the tumor microenvironment. Since the oncogenic factor of FOXM1 is highly activated through increasing demethylation of H3 lysine 79 (H3K79me2) in bone marrow-derived dendritic cells (BMDCs), this transcription factor can suppress maturation phenotypes and function of BMDCs by activation of Wnt5a noncanonical signaling. It has been reported that exogenous expression of Wnt5a also inhibits maturation phenotypes of BMDC through suppression of FOXM1 promoter demethylation [64].

## 6. Conclusion

**Figure 1:**
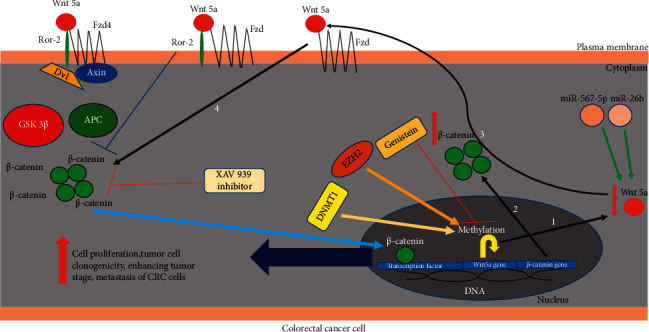
Effects of pharmacological and biological regulators on the expression levels of Wnt5a through changes of histone-modification and methylation in the CRC.

This review summarizes the recent findings on the clinical importance and function of Wnt5a in the pathogenesis of colorectal cancer (CRC) (Tables 1 and 2). The oncogenic and tumor suppressor effects of Wnt5a on the different stages of CRC suggest that Wnt5a noncanonical signaling can be used as an attractive candidate target for this disease. However, for considering the ligand as an appropriate therapeutic target, it is necessary to precisely examine and eventually determine action mechanisms of Wnt5a along with other inhibitors and stimulator markers during CRC progression. So, further studies can be considered based on using Wnt5a antagonists as new therapeutic strategies to allowbetter understanding and management of this disease (Figure 1).

## Figures and Tables

**Table 1:** Inhibitory effects of Wnt5a during CRC development.

Factor	Wnt5a expression level	Wnt5a function	Mechanism	CRC sample type	Reference
Methylation	Low	Tumor suppressor	Stimulation of tumor cell clonogenicity by upregulation of *β*-catenin and overactivation of Wnt *β*-catenin signaling	CRC cell lines	[44]
Ectopic model	High	Tumor suppressor	Inhibition of cell proliferation and metastasis via suppression of EMT, Wnt canonical, and overactivation of Wnt/Ca^2+^ signaling	HCT116	[45]
Unmethylation	High	Tumor suppressor	Increasing inhibitory effect of 5-FU	LoVo, SW480, HCT116, and SW620	[46]
Vitamin D intake	High	Tumor suppressor	Inhibition of cell proliferation through enhancing levels of Wnt5a methylation	Tissue	[47]
Genistein	High	Tumor suppressor	Suppression of cell proliferation via reducing levels of CpG island methylation of Wnt5a	SW1116	[48]
Trichostatin A (TSA)/sodium butyrate (NaBt)	High	Tumor suppressor	Stimulation of levels of Wnt5a expression by decreasing acetylation levels of histones including H3Ac, H4Ac, and H3K4me2	SW620 and SW480	[49]
EZH2	Low	Tumor suppressor	Enhancing cell proliferation via hypermethylation of Wnt5a promotes H3K27 and overactivation of TGF-*β* signaling	SW480	[50]
Recombinant Wnt5a (rWnt5a)/Foxy-5	High	Tumor suppressor	Inhibition of cell proliferation and stimulation of cell differentiation by overexpression of 15-PGDH and inactivation of Wnt *β*-catenin	HT-29 and Caco-2	[51]
CaSR	High	Tumor suppressor	Suppression of cell proliferation via inactivation of Wnt *β*-catenin and overexpression of Ror2 and Siah-2	HT-29	[52]
LPS	High	Tumor suppressor	Reduction of TNF-*α*-induced Wnt3a signaling via decreasing expression levels of TNFR1 and increasing CaSR and Ror2	CaSR-HEK, RAW264.7, and HT-29	[53]
miR-567-5p	Low	Tumor suppressor	Stimulation of cell proliferation by upregulation of *β*-catenin and downregulation of Wnt5a	SW480	[55]
pcDNA-Wnt5a/XAV939 inhibitor	High	Tumor suppressor	Inhibition of cell viability, migration, and invasion through suppression of miR-567-5p activity and inactivation of Wnt *β*-catenin signaling	SW480	[55]
miR-26b	Low	Tumor suppressor	Stimulation of CRC cells metastasis via enhancing EMT and downregulation of Wnt5a and PTEN	Caco-2, DLD1	[56]
Methylation	Low	Tumor suppressor	Increasing MSI tumors features	Tissue	[57]
DNMT1	Low	Tumor suppressor	Enhancing tumor stage	Tissue	[58]

**Table 2:** Stimulatory effects of Wnt5a during CRC development.

Factor	Wnt5a expression level	Wnt5a function	Mechanism	CRC sample type	Reference
TAM	High	Oncogenic	Stimulation of cell proliferation and migration through activation of CaMKǀǀ-ERK signaling	HCT116, DLD1, and tissue	[59]
Autocrine effect of Wnt5a	High	Oncogenic	Inducing cell proliferation, migration, and invasion via stimulation of M2 polarization of TAM and enhancing secreted IL-10 in TAMs as well as activation of CaMKǀǀ-ERK1/2-STAT3-mediated IL-10-secreting TAMs	HCT116, DLD1	[60]
TrpC5	High	Oncogenic	Decreasing differentiation and increasing stemness CRC cells by upregulation of Wnt5a and *β*-catenin	SW480, HT-29, SW620, DLD1, HCE8693, and RKO	[62]
DSS-induced CAC	High	Oncogenic	Overexpression of Wnt5a, Ror2, Fzd-2, and -5 induced by DSS lead to promote survival and proliferation through increasing CAMK2*γ* and activation of STAT3 signaling	IEC	[63]
FOXM1	High	Oncogenic	Inhibition of maturation phenotypes and functions of BMDCs by increasing FOXM1 H3K79me2 demethylation-induced activation of Wnt5a signaling	BMDC	[64]

## Data Availability

There are no raw data associated with this review.

## References

[1] Logan C. Y., Nusse R. (2004). The Wnt signaling pathway in development and disease. *Annual Review of Cell and Developmental Biology*.

[2] Wodarz A., Nusse R. (1998). Mechanisms of Wnt signaling in development. *Annual Review of Cell and Developmental Biology*.

[3] Ghosh N., Hossain U., Mandal A., Sil P. C. (2019). The Wnt signaling pathway: a potential therapeutic target against cancer. *Annals of the New York Academy of Sciences*.

[4] Teo J.-L., Kahn M. (2010). The Wnt signaling pathway in cellular proliferation and differentiation: a tale of two coactivators. *Advanced Drug Delivery Reviews*.

[5] Eubelen M., Bostaille N., Cabochette P. (2018). A molecular mechanism for Wnt ligand-specific signaling. *Science*.

[6] Pai S. G., Carneiro B. A., Mota J. M. (2017). Wnt/beta-catenin pathway: modulating anticancer immune response. *Journal of Hematology & Oncology*.

[7] Voronkov A., Krauss S. (2013). Wnt/beta-catenin signaling and small molecule inhibitors. *Current Pharmaceutical Design*.

[8] Li V. S. W., Ng S. S., Boersema P. J. (2012). Wnt signaling through inhibition of *β*-catenin degradation in an intact Axin1 complex. *Cell*.

[9] Kolligs F. T., Bommer G., Göke B. (2002). Wnt/beta-catenin/tcf signaling: a critical pathway in gastrointestinal tumorigenesis. *Digestion*.

[10] Doumpas N., Lampart F., Robinson M. D., Lentini A., Nestor C. E., Cantù C. (2019). TCF/LEF dependent and independent transcriptional regulation of Wnt/*β*‐catenin target genes. *The EMBO Journal*.

[11] Widelitz R. (2005). Wnt signaling through canonical and non-canonical pathways: recent progress. *Growth Factors*.

[12] Kohn A. D., Moon R. T. (2005). Wnt and calcium signaling: *β*-catenin-independent pathways. *Cell Calcium*.

[13] Habas R., Kato Y., He X. (2001). Wnt/frizzled activation of Rho regulates vertebrate gastrulation and requires a novel Formin homology protein Daam1. *Cell*.

[14] Marlow F., Topczewski J., Sepich D., Solnica-Krezel L. (2002). Zebrafish Rho kinase 2 acts downstream of Wnt11 to mediate cell polarity and effective convergence and extension movements. *Current Biology*.

[15] Li L., Yuan H., Xie W. (1999). Dishevelled proteins lead to two signaling pathways. *Journal of Biological Chemistry*.

[16] Weiser D. C., Pyati U. J., Kimelman D. (2007). Gravin regulates mesodermal cell behavior changes required for axis elongation during zebrafish gastrulation. *Genes & Development*.

[17] Gordon M. D., Nusse R. (2006). Wnt signaling: multiple pathways, multiple receptors, and multiple transcription factors. *Journal of Biological Chemistry*.

[18] Malbon C. C., Kühl M., Sheldahl L. C., Moon R. T. (2000). Ca2+/calmodulin-dependent protein kinase II is stimulated by Wnt and frizzled homologs and promotes ventral cell fates in Xenopus. *Journal of Biological Chemistry*.

[19] Hogan P. G., Chen L., Nardone J., Rao A. (2003). Transcriptional regulation by calcium, calcineurin, and NFAT. *Genes & Development*.

[20] Feske S., Okamura H., Hogan P. G., Rao A. (2003). Ca2+/calcineurin signalling in cells of the immune system. *Biochemical and Biophysical Research Communications*.

[21] De A. (2011). Wnt/Ca2+ signaling pathway: a brief overview. *Acta Biochimica et Biophysica Sinica*.

[22] Semenov M. V., Habas R., MacDonald B. T., He X. (2007). SnapShot: noncanonical Wnt signaling pathways. *Cell*.

[23] Leris A. C., Roberts T. R., Jiang W. G., Newbold R. F., Mokbel K. (2005). WNT5A expression in human breast cancer. *Anticancer Research*.

[24] Mikels A. J., Nusse R. (2006). Purified Wnt5a protein activates or inhibits *β*-catenin-TCF signaling depending on receptor context. *PLoS Biology*.

[25] Abedini A., Zamberlam G., Boerboom D., Price C. A. (2015). Non-canonical WNT5A is a potential regulator of granulosa cell function in cattle. *Molecular and Cellular Endocrinology*.

[26] Fu H.-D., Wang B.-K., Wan Z.-Q., Lin H., Chang M.-L., Han G.-L. (2016). Wnt5a mediated canonical Wnt signaling pathway activation in orthodontic tooth movement: possible role in the tension force-induced bone formation. *Journal of Molecular Histology*.

[27] Civenni G., Holbro T., Hynes N. E. (2003). Wnt1 and Wnt5a induce cyclin D1 expression through ErbB1 transactivation in HC11 mammary epithelial cells. *EMBO Reports*.

[28] Nemeth M. J., Topol L., Anderson S. M., Yang Y., Bodine D. M. (2007). Wnt5a inhibits canonical Wnt signaling in hematopoietic stem cells and enhances repopulation. *Proceedings of the National Academy of Sciences*.

[29] Bisson J. A., Mills B., Paul Helt J.-C., Zwaka T. P., Cohen E. D. (2015). Wnt5a and Wnt11 inhibit the canonical Wnt pathway and promote cardiac progenitor development via the Caspase-dependent degradation of AKT. *Developmental Biology*.

[30] Topol L., Jiang X., Choi H., Garrett-Beal L., Carolan P. J., Yang Y. (2003). Wnt-5a inhibits the canonical Wnt pathway by promoting GSK-3-independent *β*-catenin degradation. *Journal of Cell Biology*.

[31] Zhu N., Qin L., Luo Z., Guo Q., Yang L., Liao D. (2014). Challenging role of Wnt5a and its signaling pathway in cancer metastasis (review). *Experimental and therapeutic medicine*.

[32] Torres M. A., Yang-Snyder J. A., Purcell S. M., DeMarais A. A., McGrew L. L., Moon R. T. (1996). Activities of the Wnt-1 class of secreted signaling factors are antagonized by the Wnt-5A class and by a dominant negative cadherin in early Xenopus development. *Journal of Cell Biology*.

[33] Bauer M., Bénard J., Gaasterland T., Willert K., Cappellen D. (2013). WNT5A encodes two isoforms with distinct functions in cancers. *PLoS One*.

[34] Asem M., Buechler S., Wates R., Miller D., Stack M. (2016). Wnt5a signaling in cancer. *Cancers*.

[35] Prasad C. P., Manchanda M., Mohapatra P., Andersson T. (2018). WNT5A as a therapeutic target in breast cancer. *Cancer and Metastasis Reviews*.

[36] Kobayashi Y., Kadoya T., Amioka A. (2018). Wnt5a-induced cell migration is associated with the aggressiveness of estrogen receptor-positive breast cancer. *Oncotarget*.

[37] Trifa F., Karray-Chouayekh S., Jmal E. (2013). Loss of WIF-1 and Wnt5a expression is related to aggressiveness of sporadic breast cancer in Tunisian patients. *Tumor Biology*.

[38] Thiele S., Göbel A., Rachner T. D. (2015). WNT5A has anti-prostate cancer effects in vitro and reduces tumor growth in the skeleton in vivo. *Journal of Bone and Mineral Research*.

[39] Hibi K., Mizukami H., Goto T. (2009). WNT5A gene is aberrantly methylated from the early stages of colorectal cancers. *Hepato-Gastroenterology*.

[40] Abdelmaksoud-Dammak R., Miladi-Abdennadher I., Saadallah-Kallel A. (2014). Downregulation of WIF-1 and Wnt5a in patients with colorectal carcinoma: clinical significance. *Tumor Biology*.

[41] Vermorken J., Cervantes A., Morsing P. (2019). A randomized, multicenter, open-label controlled phase 2 trial of Foxy-5 as neoadjuvant therapy in patients with WNT5A negative colon cancer. *Annals of Oncology*.

[42] Proto M. C., Fiore D., Piscopo C. (2017). Inhibition of Wnt/*β*-Catenin pathway and histone acetyltransferase activity by rimonabant: a therapeutic target for colon cancer. *Scientific Reports*.

[43] Ying Y., Tao Q. (2009). Epigenetic disruption of the WNT/*β*-catenin signaling pathway in human cancers. *Epigenetics*.

[44] Chan A. T., Sung J. J., Tao Q. (2008). WNT5A exhibits tumor-suppressive activity through antagonizing the Wnt/B-catenin signaling, and is frequently methylated in colorectal cancer. *Clinical Cancer Research*.

[45] Cheng R., Sun B., Liu Z. (2014). Wnt5a suppresses colon cancer by inhibiting cell proliferation and epithelial-mesenchymal transition. *Journal of Cellular Physiology*.

[46] Jiang G., Lin J., Wang W., Sun M., Chen K., Wang F. (2017). WNT5A promoter methylation is associated with better responses and longer progression-free survival in colorectal cancer patients treated with 5-fluorouracil-based chemotherapy. *Genetic Testing and Molecular Biomarkers*.

[47] Rawson J. B., Sun Z., Dicks E. (2012). Vitamin D intake is negatively associated with promoter methylation of the Wnt antagonist GeneDKK1in a large group of colorectal cancer patients. *Nutrition and Cancer*.

[48] Wang Z., Chen H. (2010). Genistein increases gene expression by demethylation of WNT5a promoter in colon cancer cell line SW1116. *Anticancer Research*.

[49] Li Q., Chen H. (2012). Silencing of Wnt5a during colon cancer metastasis involves histone modifications. *Epigenetics*.

[50] Tao J., Shi L., Huang L. (2017). EZH2 is involved in silencing of WNT5A during epithelial-mesenchymal transition of colon cancer cell line. *Journal of Cancer Research and Clinical Oncology*.

[51] Mehdawi L. M., Prasad C. P., Ehrnström R., Andersson T., Sjölander A. (2016). Non-canonical WNT5A signaling up-regulates the expression of the tumor suppressor 15-PGDH and induces differentiation of colon cancer cells. *Molecular Oncology*.

[52] MacLeod R. J., Hayes M., Pacheco I. (2007). Wnt5a secretion stimulated by the extracellular calcium-sensing receptor inhibits defective Wnt signaling in colon cancer cells. *American Journal of Physiology-Gastrointestinal and Liver Physiology*.

[53] Kelly J. C., Lungchukiet P., MacLeod R. J. (2011). Extracellular calcium-sensing receptor inhibition of intestinal epithelialTNF signaling requires CaSR-mediated Wnt5a/Ror2 interaction. *Frontiers in Physiology*.

[54] Miyoshi H., Ajima R., Luo C. T., Yamaguchi T. P., Stappenbeck T. S. (2012). Wnt5a potentiates TGF-*β* signaling to promote colonic crypt regeneration after tissue injury. *Science*.

[55] Luo J., Liu L., Shen J. (2021). miR-576-5p promotes epithelial-to-mesenchymal transition in colorectal cancer by targeting the Wnt5a-mediated Wnt/*β*-catenin signaling pathway. *Molecular Medicine Reports*.

[56] Fan D., Lin X., Zhang F. (2018). MicroRNA 26b promotes colorectal cancer metastasis by downregulating phosphatase and tensin homolog and wingless-type MMTV integration site family member 5A. *Cancer Science*.

[57] Rawson J. B., Mrkonjic M., Daftary D. (2011). Promoter methylation of Wnt5a is associated with microsatellite instability and BRAF V600E mutation in two large populations of colorectal cancer patients. *British Journal of Cancer*.

[58] Samaei N. M., Yazdani Y., Alizadeh-Navaei R., Azadeh H., Farazmandfar T. (2014). Promoter methylation analysis of WNT/*β*-catenin pathway regulators and its association with expression of DNMT1 enzyme in colorectal cancer. *Journal of Biomedical Science*.

[59] Liu Q., Song J., Pan Y. (2020). Wnt5a/CaMKII/ERK/CCL2 axis is required for tumor-associated macrophages to promote colorectal cancer progression. *International Journal of Biological Sciences*.

[60] Liu Q., Yang C., Wang S. (2020). Wnt5a-induced M2 polarization of tumor-associated macrophages via IL-10 promotes colorectal cancer progression. *Cell Communication and Signaling: CCS*.

[61] Bakker E. R. M., Das A. M., Helvensteijn W. (2013). Wnt5a promotes human colon cancer cell migration and invasion but does not augment intestinal tumorigenesis in Apc 1638N mice. *Carcinogenesis*.

[62] Chen Z., Tang C., Zhu Y. (2017). TrpC5 regulates differentiation through the Ca2+/Wnt5a signalling pathway in colorectal cancer. *Clinical Science*.

[63] Ma X., Meng Z., Jin L. (2017). CAMK2*γ* in intestinal epithelial cells modulates colitis-associated colorectal carcinogenesis via enhancing STAT3 activation. *Oncogene*.

[64] Zhou Z., Chen H., Xie R. (2019). Epigenetically modulated FOXM 1 suppresses dendritic cell maturation in pancreatic cancer and colon cancer. *Molecular oncology*.

